# Egyptian practical guidance in hypertriglyceridemia management 2021

**DOI:** 10.1186/s43044-021-00235-9

**Published:** 2021-12-20

**Authors:** Hesham Salah El Din Taha, Hossam Kandil, Nabil Farag, Abbas Oraby, Magdy El Sharkawy, Fouad Fawzy, Hossam Mahrous, Juliette Bahgat, Mina Samy, Mohamed Aboul, Mostafa Abdrabou, Mirna Mamdouh Shaker

**Affiliations:** 1 Department of Cardiology, Faculty of Medicine, Cairo University, 27 Nafezet Sheem El Shafae St Kasr Al Ainy, Cairo, 11562 Egypt; 2 Ain-Shams University, Cairo, Egypt; 3 Suez Canal University, Ismailia, Egypt

**Keywords:** Hypertriglyceridemia, Dyslipidemia, Practical guidance, Atherosclerotic cardiovascular disease

## Abstract

Hypertriglyceridemia (HTG) is a very common, yet underappreciated problem in clinical practice. Elevated triglyceride (TG) levels are independently associated with atherosclerotic cardiovascular disease (ASCVD) risk. Furthermore, severe HTG may lead to acute pancreatitis. Although LDL-guided statin therapy has improved ASCVD outcomes, residual risk remains. Recent trials have demonstrated that management of high TG levels, in patients already on statin therapy, reduces the rate of major vascular events. Few guidelines were issued, providing important recommendations for HTG management strategies. The goal of treatment is to reduce the risk of ASCVD and acute pancreatitis. The management stands on lifestyle modification, detection of secondary causes of HTG and pharmacological therapy, when indicated. In this guidance we review the causes and classification of HTG and summarize the current methods for risk estimation, diagnosis and treatment. The present guidance provides a focused update on the management of HTG, outlined in a simple user-friendly format, with an emphasis on the latest available data.

## Background

Hypertriglyceridemia (HTG) prevalence in adults varies between 10 and 29% according to the studied population [1]. HTG is classified as primary; due to genetic causes, or may be secondary to particular diseases, drugs or metabolic disorders [2].

Dyslipidemia itself usually causes no symptoms but can lead to symptomatic vascular disease, including coronary artery disease (CAD), cerebrovascular disease and peripheral arterial disease (PAD). In hereditary lipid disorders, a patient may present with the variants of cutaneous xanthoma. Eruptive xanthomas at pressure sites on the elbows, buttocks, and knees may be indicative of hypertriglyceridemia. High levels of TG (> 500 mg/dL) can cause *acute pancreatitis*. Higher TG levels can also cause dyspnea, hepatosplenomegaly lipemia retinalis and neurological manifestations such as confusion, transient memory loss, paresthesias and rarely, focal neurologic deficits. Extremely high lipid levels also give a lactescent (milky) appearance to blood plasma [3–5].

HTG is an underappreciated risk factor that is often overshadowed by low-density lipoprotein cholesterol (LDL-C) and other chronic conditions [6]. Studies have shown that despite the use of statin therapy, atherosclerotic cardiovascular disease (ASCVD) event rates remain high in patients with elevated TG [7]. The importance of lowering plasma levels of TG is currently considered an integral part of residual cardiovascular risk reduction strategies [8]. The American College of Cardiology/American Heart Association cholesterol guidelines list HTG as a risk-enhancing factor for cardiovascular disease (CVD) [9].

Dietary TGs are carried via the intestinal lymphatics in chylomicrons, while endogenous TGs produced by the liver are transported in very low density lipoproteins (VLDL) [10]. Large triglyceride-rich lipoproteins (TRL) particles such as nascent chylomicrons are unable to infiltrate the vessel wall [11]. However, cholesteryl-ester (CE)-enriched smaller TRLs (‘remnants’) can penetrate the subendothelium promoting atherogenesis through pro-inflammatory and pro-thrombotic pathways [12].

Therefore, ASCVD risk mediated by TRLs appears to be determined by the circulating concentration of apo B-containing particles rather than their TG content. An estimate for all apo B-containing lipoproteins is non-HDL-C [calculated as total cholesterol (TC) − HDL-C], hence its importance [13].

In addition, HTG is frequently associated with pathological high-density lipoprotein (HDL) particles that may add to the ASCVD risk [12].

Furthermore, HTG is believed to be causative in up to 10% of episodes of acute pancreatitis [14]. The risk of acute pancreatitis is a continuum with mild hypertriglyceridemia associated with a low long-term risk [15, 16], while more severe HTG is associated with progressively increased risk [16, 17].

We have previously published a consensus statement on the management of lipids [18]. We felt that there was a need to address an ignored and frequently overlooked part in the management of dyslipidemia which was hypertriglyceridemia. The following guidance is based on the best scientific evidence available till present and expert opinion considerations.

## Scope of the article

The goal of this article is to provide practical guidance for clinicians in situations not covered by the current lipid guidelines, in a world with rapidly evolving evidence.

There are current gaps in care for high-risk patients with mild to moderate as well as severe forms of HTG. There are also limited data on management of patients at low to moderate atherosclerotic cardiovascular risk and moderately elevated triglycerides.

The evidence from clinical trials on triglyceride risk-based agents for ASCVD risk reduction is evolving and newer medications are soon coming into play. In this document, we aim to fulfill the following aspects:

1. Definition and categories of HTG
2. Role of lifestyle intervention in patients with HTG
3. Role of statin therapy in patients with HTG
4. Triglyceride risk-based non-statin therapies; when and how to give them?
5. Providing practical algorithms for HTG management

### Definitions and classifications

Hypertriglyceridemia is defined as a fasting plasma/serum TG level > 150 mg/dL or a non-fasting TG level > 175 mg/dL. We endorse the definition proposed by the American College of Cardiology on persistent hypertriglyceridemia; Fasting triglycerides ≥ 150 mg/dL following a minimum of 4–12 weeks of lifestyle intervention, a stable dose of maximally tolerated statin therapy when indicated, as well as evaluation and management of secondary causes of hypertriglyceridemia [7].

Patients with HTG can be categorized according to their fasting TG level as shown in Table 1.

**Table 1:** Categories of hypertriglyceridemia and goals of therapy

Category of HTG	Fasting TG level (mg/dl)	Goals
Mild	150–199	Reduce ASCVD risk and risk of pancreatitis
Moderate	200–499	
Severe	≥ 500	
Very severe	≥ 1000	

### Causes of hypertriglyceridemia

HTG may be either secondary to a variety of medical conditions or medications, or primary due to genetic causes [2]. Potential secondary causes for hypertriglyceridaemia [7] are listed in Table 2.

**Table 2:** Secondary causes of hypertriglyceridemia

Secondary causes of hypertriglyceridemia [7]:	
Diseases	Uncontrolled diabetes mellitus, chronic kidney disease, nephrotic syndrome, hypothyroidism, Cushing’s disease
Metabolic and dietary disorders	Overweight/obesity, metabolic syndrome, sedentary lifestyle, alcohol abuse or alcohol excess, diets high in saturated fat, sugar, or high-glycemic index foods, total parenteral nutrition with lipid emulsions
Drugs	Propofol, beta blockers, diuretics, thiazide and loop diuretic agents, bile acid sequestrants, glucocorticosteroids, anabolic steroids, oral estrogens, tamoxifen, HIV protease inhibitors, atypical antipsychotics, tacrolimus, sirolimus, cyclosporine, interferons
Pregnancy	Especially in the third trimester

While secondary factors more frequently cause mild to moderate HTG, severe forms of HTG are more commonly primary in nature [10]. The causes of primary HTG are listed in Table 3 [19–22].

**Table 3:** Different types of familial HTG [19–22]

	FCH (Type IIB)	FCS (Type I)	MFCS (Type IV)	FHTG (Type V)	FD (Type III)
Lipoprotein change	↑VLDL, LDL	↑Chylomicrons	↑VLDL	↑VLDL, chylomicrons	↑IDL
Lipid change	↑TG, TC	↑TG	↑TG	↑TG, TC	↑TG, TC
Incidence	1/100–200	1/500,000–1,000,000	1/600–1000	1/500	1/10,000
Genetics	Polygenic (TG, LDL raising alleles)	Monogenic homozygous (autosomal recessive)^a^	Monogenic heterozygous (autosomal dominant)^a^ Polygenic	Monogenic heterozygous (autosomal dominant)^a^ Polygenic	Monogenic homozygous (defect in APOE gene)
Time of presentation	All in adulthood (earlier with secondary causes) except for FCS (type I) in childhood				
Specific for diagnosis	Combination of: ApoB > 120 mg/dL TGs > 133 mg/dL FH of premature CVD	TG > 885 mg/dl Creamy appearance of the blood Failure to thrive, Recurrent abdominal pain, nausea, vomiting Acute pancreatitis (60–80% lifetime risk) Tuberous xanthoma Lipemia retinalis Hepatosplenomegaly	TG 150–885 mg/dl or > 885 mg/dl with secondary insult Responsive to standard therapy Require an aggravating effect	TG 150–885 mg/dl Require an aggravating effect Lower risk of pancreatitis	Palmer crease xanthomas are pathognomonic

*FCH* familial combined hyperlipidemia, *FCS* familial chylomicronemia syndrome, *FD* familial dysbetalipoproteinaemia, *FHTG* familial HTG, *GPIHBP1* glycosylphosphatidylinositol-anchored high-density lipoprotein binding protein-1, *MFCS* multifactorial chylomicronemia syndrome, *LDL* low density lipoprotein, *LMF1* Lipase maturation factor 1, *LPL* lipoprotein lipase, *TC* total cholesterol, *TG* TG, *VLDL* very low-density lipoprotein, *FH* family history

^a^Defect in LPL, APOC2, APOA5, GPIHBP1 or LMF1 genes

### Who and when should we screen?

The age at which screening is recommended differs according to the level of risk as illustrated in Table 4.

**Table 4:** Recommendations for screening [18]

Primary prevention	All adults ≥ 40 years, however earlier screening at the age of 20 years can be considered
Secondary prevention	All patients with ASCVD, (e.g., CAD, CVD, PAD), or multiple major risk factors
Family history of early CVD or familial dyslipidemia	As early as the second year of life

Screening for HTG should also be considered in patients with [7]:

- Acute pancreatitis
- Suspected primary HTG:
  - Presence of tuberous xanthomas in the patient or family member
  - Family history of HTG
  - Severely elevated TG levels
  - Premature ASCVD in the patient or family member
- Secondary causes for HTG

### How should we screen?

For screening, lipid testing should include total cholesterol (TC), low-density lipoprotein cholesterol (LDL-C), high-density lipoprotein cholesterol (HDL-C), TG and non-HDL-C. It can generally be done using a non-fasting blood sample. The postprandial rise in TG is usually small, between 12 and 27 mg/dL. Fasting lipid testing is preferred in patients with metabolic syndrome, family history of premature ASCVD or genetic lipid disorders, non-fasting TG ≥ 400 mg/dL and to assess adherence to lifestyle interventions and lipid lowering medications [7, 9, 18, 23, 24].

### Management of HTG in patients without ASCVD or diabetes mellitus (DM)

**Fig. 1:**
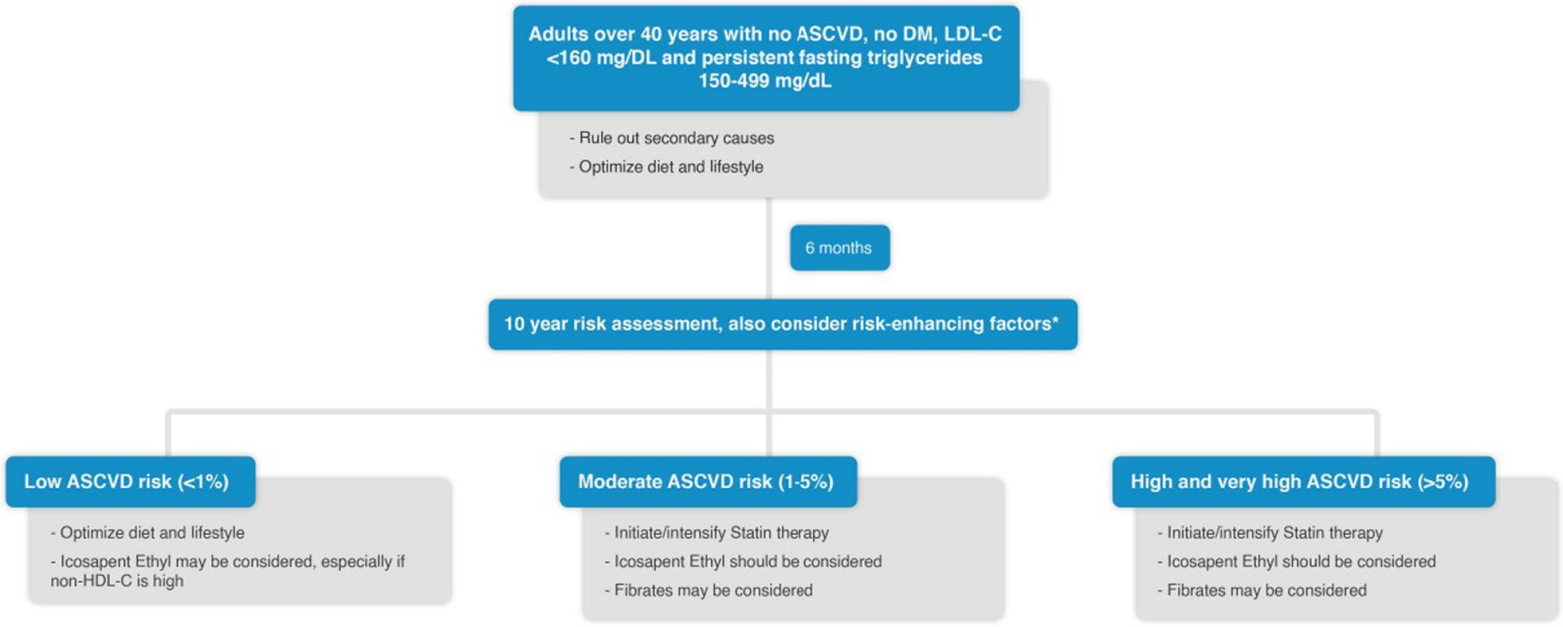
Management of HTG in patients without ASCVD or DM. *ASCVD risk-enhancing factors including family history of premature ASCVD, persistently elevated LDL-C ≥ 160 mg/dL, chronic kidney disease, metabolic syndrome, inflammatory diseases (especially rheumatoid arthritis, psoriasis), biomarkers (persistently elevated fasting triglycerides ≥ 150 mg/dL, hs-CRP ≥ 2.0 mg/L) [7].

**Fig. 2:**
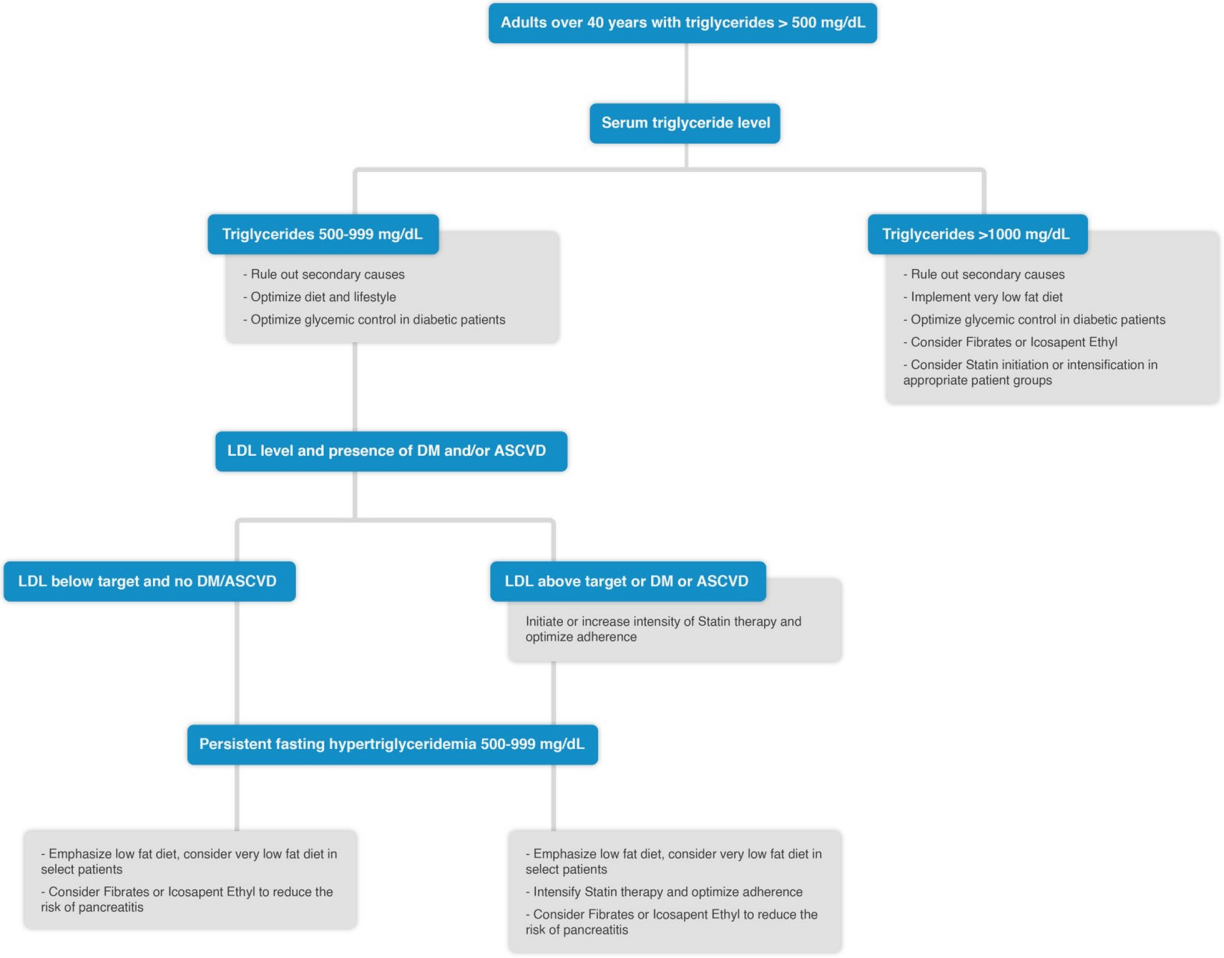
Management of HTG ≥ 500 mg/dl in patients without ASCVD or DM

Limited data are available on ASCVD risk reduction using TG risk-based non-statin therapies for primary prevention in non-diabetic patients with persistent mild to moderate HTG [7]. Management of these patients is described in Fig. 1. However, patients with more severe forms of HTG have a relatively higher incidence of acute pancreatitis [7]. Management of these patients is described in Fig. 2.

### Management of HTG in patients with ASCVD

**Fig. 3:**
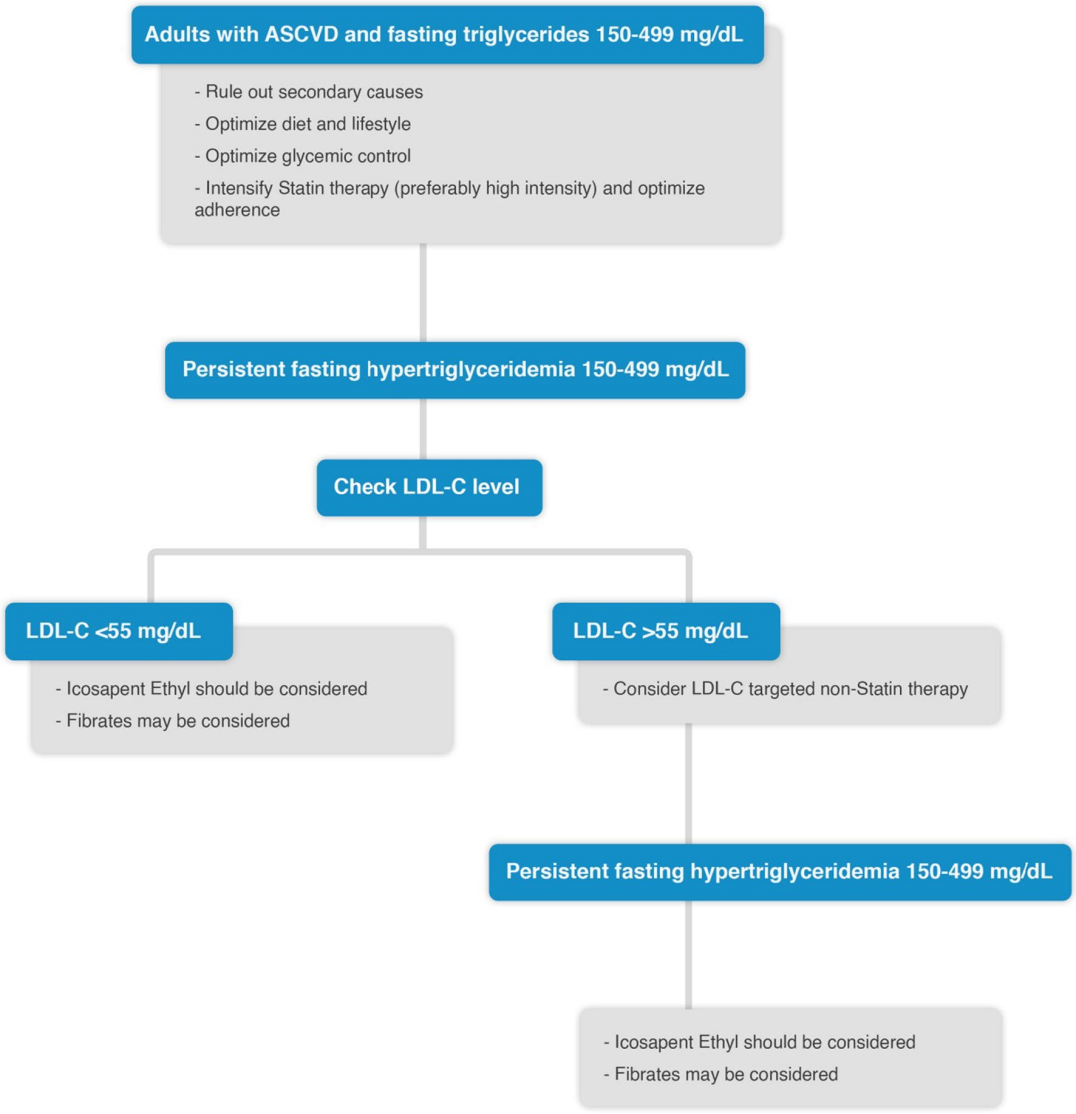
Management of HTG in patients with ASCVD

Individuals with persistent moderate to severe HTG may be at increased risk of premature CVD. Few studies have shown an independent relationship, although small, between HTG and CVD. Studies have suggested that the ApoB-containing particles and/or lipoprotein (a), rather than the TG content of TRLs and their remnants are the cause of ASCVD risk [25, 26]. Management of HTG in ASCVD patients is illustrated in Fig. 3.

### Management of HTG in patients with DM

The leading cause of mortality in diabetes mellitus is CVD. Diabetic patients have higher risk of developing ASCVD and much of this risk can be attributed to the development of atherogenic dyslipidemia, in which HTG is an essential component.

**Fig. 4:**
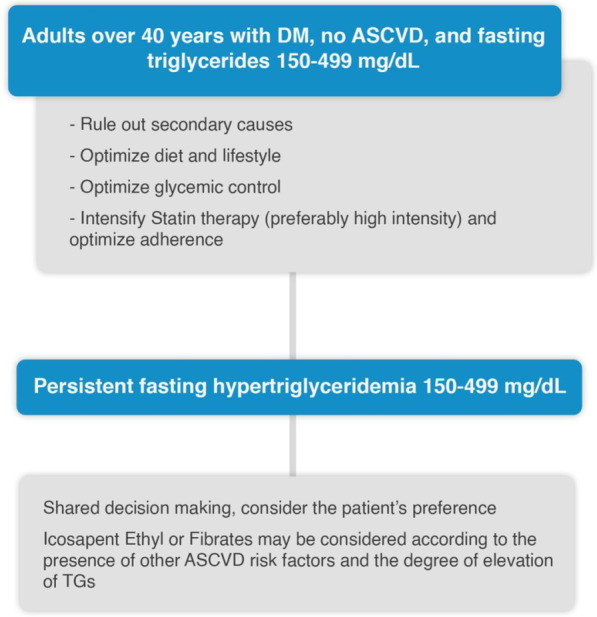
Management of HTG in diabetic patients without ASCVD

In well-controlled DM, the lipid profile may be normal, while in poorly controlled DM, the lipid profile shows a triad of HTG, increased levels of small dense LDL, and low HDL [27]. The management algorithm of HTG in diabetic patients is shown Figs. 2 and 4*.*

### Management of HTG in patients with chronic kidney disease (CKD)

HTG is frequently encountered in the setting of CKD, especially in association with nephrotic syndrome and in patients on peritoneal dialysis [28]. The exact mechanisms through which CKD causes HTG are not exactly known, however, increased catabolism of VLDL [due to increased lipoprotein lipase (LPL) inhibitors as apo-C III] and increased VLDL production have been suggested [29]. Risk factors for development of HTG in CKD include DM, the degree of glomerular filtration rate (GFR) affection, severity of proteinuria, the modality of renal replacement therapy (peritoneal dialysis more than hemodialysis), nutritional status of the patient, and the presence of other comorbidities [14].

The aim of treatment is to primarily reduce the patient’s cardiovascular (CV) risk and in patients with severe HTG, reduce the risk of pancreatitis [14]. Non-pharmacological treatment is indicated as in patients without CKD, but diet modification is not recommended in malnourished patients. Statins are the cornerstone of treatment and are recommended to all patients with non-dialysis-dependent stage 3 or higher CKD, or recipients of renal transplantation. They should not be started in dialysis-dependent patients but should be continued if already on statins before being dialysis-dependent [23]. Caveats of statin use in CKD include the need for dose adjustment of some statins and the higher risk of rhabdomyolysis when used with cyclosporine or fibrates [11]. Icosapent Ethyl should be considered if HTG persists on statin therapy [30]. Whether there is a net benefit or not on CV outcomes with fibrate use in CKD is still unknown. Fibrates are therefore not recommended for the reduction of CV risk in CKD patients [14]. They may be considered in patients with severe HTG who are at high risk of pancreatitis under specialist consultation. Fibrates should be adjusted to GFR. Fibrates may lower cyclosporine levels in patients with renal transplants [14]. Lomitapide may be considered in patients with severe HTG who are at high risk of pancreatitis, with dose adjustment to GFR [31].

### Management of HTG in patients with familial hypertriglyceridemia (FHTG)

There are five different types of FHTG classified according to the type of elevated lipoprotein and associated lipid changes including: familial combined hyperlipidemia (FCH), which is the commonest, familial chylomicronemia syndrome (FCS), familial dysbetalipoproteinaemia (FD), familial HTG (FHTG), and multifactorial chylomicronemia syndrome (MFCS) (Table 3) [19–22].

Pharmacologic management is frequently needed in FHTG to prevent pancreatitis and/or reduce risk of CVD. Medications commonly used for TG lowering are icosapent ethyl, fibrates, and Niacin [32]. In individuals who are unresponsive to treatment, plasmapheresis has been used.

There has been considerable interest in developing new TG lowering agents, especially for treatment of FHTG, using approaches targeting LPL and its regulators because of the central role they play in TRL metabolism. Volanesorsen is an antisense oligonucleotide that binds to apolipoprotein C3 (APOC3) mRNA preventing APOC3 translation. It has been shown to lower circulating APOC3 levels by 70–90% and TG levels by 56–86% in clinical trials involving both FCS and non-FCS patients with moderate to severe HTG. A reduction in the number of episodes of pancreatitis was also reported. The monoclonal antibody evinacumab that blocks the action of angiopoietin-like protein 3 (ANGPTL3) has been found to lower TG levels up to 80% in patients with mild to moderate HTG, as well as LDL-C levels [33, 34].

### Lifestyle interventions

HTG is closely associated with a sedentary lifestyle and visceral adiposity. Weight loss is considered the most effective lifestyle intervention to lower TG levels; the effect of weight loss on lowering TGs is variable [35]. Five to ten percent reduction in body weight is associated with a 20% decrease in TG [34]. Higher-fat, lower carbohydrate diets have better effect on TG levels compared with lower-fat, higher carbohydrate diets [36]. The reduction in TG was greater for the very low carbohydrate regimens (in which carbohydrates represent < 10% of calories) [37]. A higher-protein (31% of caloric intake) versus a standard-protein (18% of caloric intake) weight-loss diet results in more weight loss and TG lowering [38].

There are different types of fasting, as shown in Table 5. Weight loss and greater TG lowering were noticed with alternate day fasting. A summary of nutrition recommendations for patients with HTG is shown in Table 6.

**Table 5:** Types of fasting [34–38]

Alternate day fasting	3 to 4 days/week, consumption of < 25% of energy needs during a 24-h period
Periodic fasting	Fasting 1 or 2 days/week
Time restricted eating	Food intake is limited to a specific window of time each day

**Table 6:** Summary of nutrition recommendations for patients with HTG [7, 34–38]

Nutrient	Encourage	Limit/restrict	Stop completely
Sugar-sweetened beverages		If TG < 500 mg/dl	If TG > 500 mg/dl
Fruits	Can be included but individualize 3–4 servings per day	1 serving per day if TG > 1000	
Vegetables	Encourage most vegetables	Vegetables with high glycemic index (carrots, potatoes,..)	Canned vegetables Vegetable juices
Legumes	Encourage		
Fish—sea food	Encourage fatty or lean fish at least 2 servings/week		
Poultry—lean meats	Encourage instead of red meat		
Dairy products		Limit full-fat dairy	Sugar-sweetened dairy products Full fat dairy if TG > 1000 mg/dl
Fiber-rich whole grains	Encourage 4–6 servings per day		
Nuts and peanuts	Encourage in moderation	Limit if TG > 1000 mg/dl	
Desserts		May be used occasionally if TG 500–999 mg/dl	Avoid if TGs > 1000 mg/dl

Alcohol is an important cause of elevated TG levels; patients with HTG are advised to abstain completely.

Physical activity and exercise reduce TG levels by 7–30%; the response is variable depending on the type, duration, and intensity of activity. In addition, smoking cessation should be advocated as a part of lifestyle intervention.

### Pharmacological management [32]

Classes of medications commonly used in the management of HTG include omega-3 fatty acids, fibric acid derivatives and niacin. High doses of a strong statin (simvastatin, atorvastatin, rosuvastatin) also lower TG by up to 30%. The effect of different drugs on different lipid components is illustrated in Table 7.

**Table 7:** Lipid effects of different drugs:

Drug	Lipid effects
Fenofibrate	LDL ↓: 20.6% (145 mg) HDL ↑: 11% (145 mg) TG ↓: 23.5–50.6% (greatest drop is in patients with highest TG) (145 mg)
IPE	HDL ↑: 9.1% (4 g/day) TG ↓: 45% (4 g/day)
Niacin	LDL ↓: 14–17% (2 g/day) HDL ↑: 22–26% (2 g/day) TG ↓: 20–50%
Ezetimibe	LDL ↓: 18% (10 mg/day) HDL ↑: 1% (10 mg/day) TG ↓: 8%
Statins	LDL ↓: 30–50% (dose, and drug dependent) HDL ↑: 1–10% TG ↓: 10–30%

### Fibrates [39]

Fibrates are commonly used to treat HTG. They increase the activity of LPL through peroxisome proliferator activated receptor (PPAR) alpha receptor agonist action, which hydrolyzes TG in TRL.

Fibrates may be associated with a reversible increase in serum creatinine; therefore the dose should be lowered in patients with mild-to-moderate renal disease (about one third of the usual dose) and should be avoided in severe renal impairment. Risk for myopathy/ rhabdomyolysis increases with renal impairment or concurrent use of HMG-CoA reductase inhibitors. Rarely, severe anemia, leukopenia, thrombocytopenia may occur so periodic blood counts are recommended.

### Omega 3 fatty acids [40]

Icosapent ethyl and docosahexaenoic acid (DHA) are the active constituents of the omega 3 fatty acids. Higher doses of icosapent ethyl (2–4 g) significantly reduce the levels of TGs. Omega 3 fatty acids reduce hepatic VLDL-TG synthesis and secretion. They also increase plasma LPL activity that in turn enhances TG clearance from circulating VLDL particles. Caution should be exercised in patients allergic to fish. Recurrent atrial fibrillation or flutter has been reported with administration of high doses. Omega 3 fatty acids may increase liver alanine transaminase (ALT), therefore it should be monitored. They may prolong bleeding time; therefore, caution is needed in patients at high bleeding risk.

### Niacin [32]

Niacin reduces VLDL synthesis, and may increase chylomicron TG removal from plasma. Caution is used in patients with gout, DM, gall bladder disease, CVD, renal or hepatic impairment, if the patient is taking anticoagulants or HMG-CoA reductase inhibitors or if symptoms of myopathy occur (monitor creatine phosphokinase).

### Statins [32]

Statins are competitive inhibitors of the activity of HMG-CoA reductase enzyme. This results in the reduction of the levels of LDL. Statins reduce TG possibly through upregulation of the VLDL uptake by hepatocytes, as well as a reduction in the production rate of VLDLs. Statin-associated muscle symptoms (SAMs) may occur in 10–15%. Liver function tests should be checked at baseline, and when clinically indicated.

## Conclusions

Management algorithms proposed in this document for the management of hypertriglyceridemia are simple, cover the clinical situations most commonly observed in routine practice and are designed to improve the quality of care. In all patients with HTG, risk stratification is mandatory and an attempt should be made to identify a cause or association. All patients with HTG should have an assessment of their LDL-C and non-HDL-C levels. The goal of therapy is to reduce ASCVD risk, as well as risk of pancreatitis. The first step of treatment involves implementing lifestyle changes and management of secondary causes, if present. Proper control of other ASCVD risk factors is recommended. Statins are advised as first line agent for ASCVD risk reduction. For patients with persistently elevated TG levels after non-pharmacologic interventions and despite maximally tolerated LDL-guided therapy, we suggest introducing additional drug therapy when indicated. Pharmacological management in persistent, as well as severe HTG, is centered on omega-3 fatty acid therapy and fenofibrate.

## Future directions

HTG and its impact on ASCVD remain an under-explored frontier in lipidology. Research is needed to uncover the molecular basis and the dynamics of the different atherogenic particles in this disease process.

A risk stratification algorithm for acute pancreatitis in patients with severe HTG is needed.

More RCT-based evidence is needed for the use of fibrates to reduce CV risk in various subgroups e.g.: CKD patients and the elderly.

More data regarding the efficacy of IPE in reducing CV risk without concomitant statin use (e.g.: in patients intolerant to statins) is needed.

Further safety and efficacy data on novel drugs acting on existing pharmacological targets (e.g.: the novel selective PPAR alpha modulator-pemafibrate), or novel pharmacological targets (e.g.: apoC-III and ANGPTL-3) is needed.

## Data Availability

The dataset supporting the results and conclusions of this article will be available from the corresponding author on request.

## Abbreviations

ACC: American College of Cardiology
ANGPTL3: Angiopoietin-like protein 3
ApoB: Apolipoprotein-B
ApoC: Apolipoprotein-C
ASCVD: Atherosclerotic cardiovascular disease
CAD: Coronary artery disease
CE: Cholesteryl-ester
CKD: Chronic kidney disease
CV: Cardiovascular
CVD: Cardiovascular disease
CYP2CA: Cytochrome-P 2CA
DGAT: Diacylglycerol acyltransferase
DM: Diabetes mellitus
FCH: Familial combined hyperlipidemia
FCS: Familial chylomicronemia syndrome
FD: Familial dyslipoproteinemia
FHTG: Familial hypertriglyceridemia
GFR: Glomerular filtration rate
GPIHBP1: Glycosylphosphatidylinositol-anchored high density lipoprotein binding protein-1
HDL-C: High density lipoprotein-cholesterol
HIV: Human immunodeficiency virus
HMG-CoA: Hydroxymethylglutaryl coenzyme-A
Hs-CRP: Highly-sensitive C-reactive protein
HTG: Hypertriglyceridemia
IDL-C: Intermediate density lipoprotein-cholesterol
IPE: Icosapent ethyl
LDL-C: Low density lipoprotein-cholesterol
LMF1: Lipase maturation factor 1
LPL: Lipoprotein lipase
MCT: Medium-chain triglyceride
MFCS: Multifactorial chylomicronemia syndrome
mRNA: Messenger ribonucleic acid
PPAR: Peroxisome proliferator activated receptor
PTT: Partial thromboplastin time
SAM: Statin-associated muscle symptom
SPPARMa: Selective PPAR alpha modulator
T1DM: Type-1 diabetes mellitus
T2DM: Type-2 diabetes mellitus
TC: Total cholesterol
TG: Triglyceride
TRL: Triglyceride-rich lipoprotein
VLDL-C: Very low density lipoprotein-cholesterol
